# Primary and recurrent diffuse astrocytomas: genomic profile comparison reveals acquisition of biologically relevant aberrations

**DOI:** 10.1186/s13039-016-0222-3

**Published:** 2016-02-09

**Authors:** Halka Lhotska, Zuzana Zemanova, Hana Cechova, Sarka Ransdorfova, Karla Svobodova, Filip Kramar, Zdenek Krejcik, Kyra Michalova

**Affiliations:** Center of Oncocytogenetics, Institute of Medical Biochemistry and Laboratory Diagnostics, General University Hospital and 1st Faculty of Medicine, Charles University in Prague, U Nemocnice 449/2, 128 08 Prague 2, Czech Republic; Institute of Hematology and Blood Transfusion, Nemocnice 2094/1, 128 20 Prague 2, Czech Republic; Department of Neurosurgery, Central Military Hospital and 1st Faculty of Medicine, Charles University, U Vojenske nemocnice 1200, 169 02 Prague 6, Czech Republic

**Keywords:** Diffuse astrocytoma, MutL homolog 3, Isocitrate dehydrogenase 1, Clonality

## Abstract

**Background:**

Diffuse astrocytomas are characterized by their highly variable biological behavior. The possibility that tumors develop novel aberrations, with relevant biological properties, is often neglected. In this study, we present two cases of diffuse astrocytoma in which additional cytogenetic and epigenetic markers with potential influence on cell proliferation or differentiation were detected at relapse.

**Findings:**

The biopsies taken from the primary and recurrent tumors of two patients were analyzed with molecular methods to detect copy number variations (CNVs), gene mutations and epigenetic changes. Both cases were characterized by the R132H mutation in the isocitrate dehydrogenase 1 (*IDH1*) gene. Features typical of astrocytomas, such as copy-neutral loss of heterozygosity at 17p and the deletion of the cyclin-dependent kinase inhibitor 2A (*CDKN2A*) gene, were also detected in both cases. These markers were present in the primary and recurrent lesions. Other aberrations, predominantly deletions or amplifications of chromosomal segments and the hypermethylation of gene promoters, were detected in the recurrent lesions.

**Conclusions:**

The *IDH1* mutation was the primary event, as previously reported. According to our observations, the methylation of promoters constituted later events, which may have further disrupted cell proliferation and/or differentiation, together with additional CNVs.

## Background

Diffuse astrocytomas constitute one of the major subtypes of glial tumors, with highly variable biological behavior. In recent years, several genetic markers have been identified that predict the responses to defined therapeutic strategies, and can therefore affect the outcomes of individual patients. One example is the presence of an *IDH1* gene mutation, which confers a better overall prognosis and longer progression-free survival on patients with grade II–III astrocytoma, irrespective of treatment [1, 2]. The methylation status of the O-6-methylguanine-DNA methyltransferase (*MGMT*) promoter plays a similar role in the choice of therapy [3].

The majority of recurrent gliomas are characterized by a shared set of mutated genes and chromosomal aberrations, which probably derive from the same precursor cell [4, 5]. Therefore, many clinicians therapeutically target the molecular markers defined in the initial tumor. However, recurrent lesions appear after an asymptomatic period in the majority of patients, despite defined multidrug chemotherapy and radical surgical resection. The possibility that the tumor develops novel aberrations, with relevant biological properties, is often neglected.

In this study, we report two cases of diffuse astrocytoma in which additional cytogenetic and epigenetic markers, with potential effects on cell proliferation or differentiation, were detected at the time of relapse.

## Methods

### Interphase fluorescence in situ hybridization (I-FISH)

Dual-color I-FISH with LSI and/or CEP DNA probes (Abbott Vysis, Chicago, IL) was used to analyze copy number variations (CNVs) in the *CDKN2A* (9p21.3), *EGFR* (7p12), *PTEN* (10q23.3), *RB1* (13q14.2) and *TP53* (17p13.1) genes, in 1p and 19q regions. I-FISH was performed according to the manufacturer’s recommendations. The cut-off values were established in previous studies as 5 % for deletions and 2.5 % for amplifications [6].

### DNA isolation

The homogenized tumor tissues were used to isolate the genomic DNA (gDNA) with the DNeasy Blood and Tissue Kit (Qiagen Inc., Germantown, MD), according to the manufacturer’s protocol. gDNA obtained from peripheral blood was isolated by GenElute Blood Genomic DNA Kit (Sigma-Aldrich, St. Louis, MO).

### SNP array

The gDNA (200 ng) was hybridized onto the HumanCytoSNP-12 (v2.1) BeadChip array (Illumina, San Diego, CA), according to the manufacturer’s protocol. The array was scanned with a BeadArray Reader (Illumina) and the scan was analyzed with the BlueFuse Multi software v4.1 (Illumina). The detection limit of the SNP arrays was 15 % of cell clones.

### MLPA

Mutations in the *IDH1*/*IDH2* genes were detected with the P370 BRAF-IDH1-IDH2 probemix (MRC-Holland, Amsterdam, Netherlands). The promoter methylation of *MGMT* and six mismatch repair genes was investigated with the methylation-specific MLPA (MS-MLPA) using ME011 Mismatch Repair genes probemix (MRC-Holland). Both MLPA analyses were performed according to the manufacturer’s protocols.

### MS-PCR

MS-PCR was performed according previously published protocol [7]. Bisulfide conversion was performed with the EZ DNA Methylation-Gold™ Kit (Zymo Research, Orange, CA). The products of PCR were separated on 2 % agarose gel stained with SERVA DNA Stain Clear G (SERVA Electrophoresis GmbH, Heidelberg, Germany).

## Results

Both patients were diagnosed with astrocytoma and treated at the Department of Neurosurgery, Central Military Hospital and 1st Faculty of Medicine, Charles University, Prague, Czech Republic. The patients gave their written consent for the use of their biological material for research purposes. The tumor tissues were taken during routine neurosurgical procedures and peripheral blood was used as the negative control. All resections were characterized by I-FISH, SNP array, MLPA and MS-PCR.

**Fig. 1:**
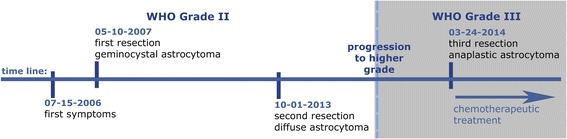
Time line representing the dates of resection and the type of treatment the case no. 1 received

Case no. 1 was a male patient who was diagnosed with gemistocytic astrocytoma (WHO grade II) at the age of 31 years. Six years later, a second radical resection (no tumor remnant was detected) was performed and the tumor was determined to be a diffuse astrocytoma (WHO grade II). A third resection (anaplastic astrocytoma WHO grade III) was performed 5 months later (Fig. 1). The tumor was localized in the left frontal lobe. The patient is currently undergoing treatment with chemotherapy and his survival is 101 months.

**Fig. 2:**
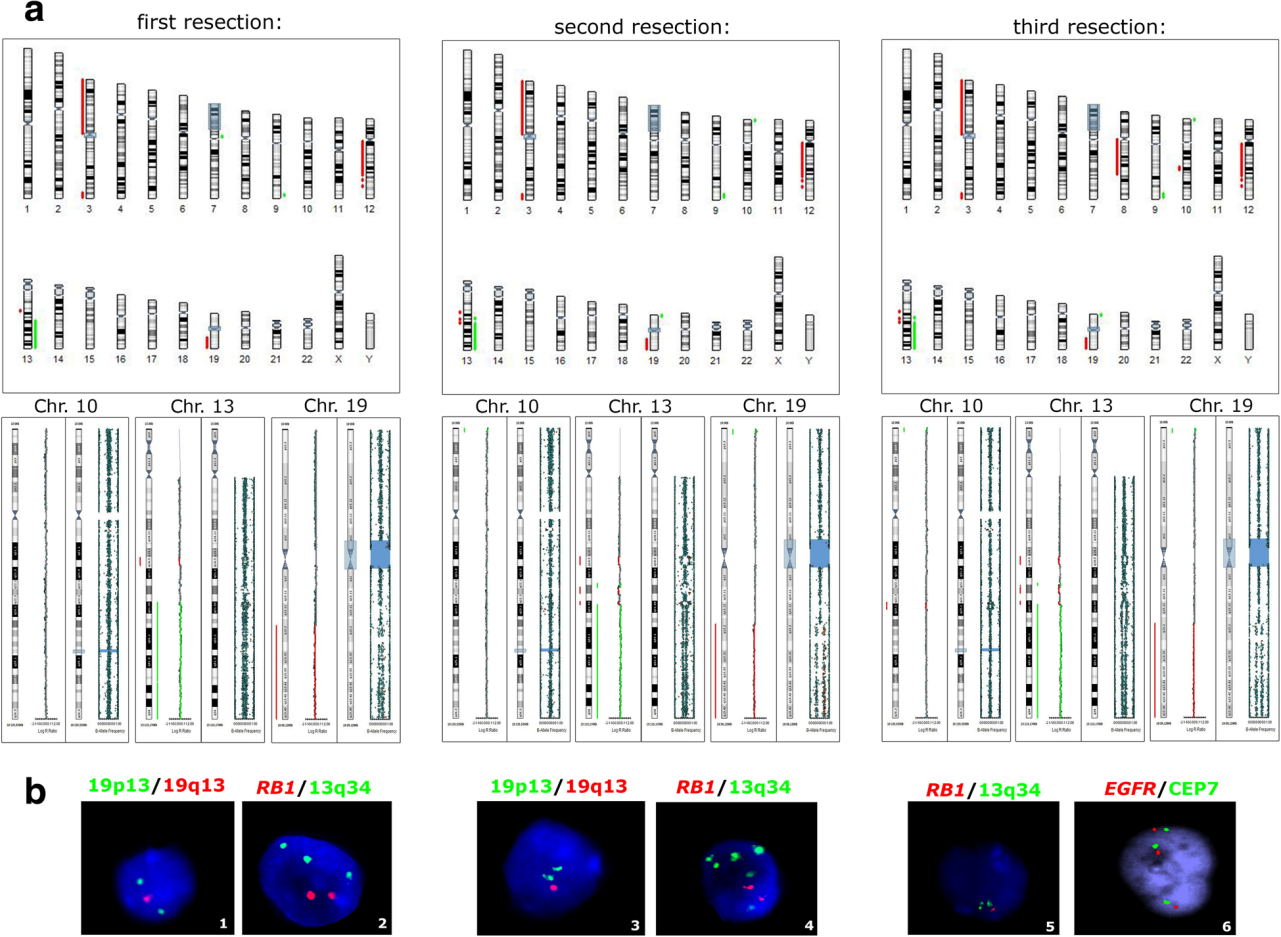
Molecular cytogenetic and epigenetic analyses of the three lesions of case no. 1. **a** Results of a SNP array for each lesion, with the selection of three chromosomes, in which additional changes were detected. Red bars indicate deletions of a chromosomal region. Green bars represent amplifications, and CN-LOH is marked with gray boxes. **b** Result of I-FISH analysis confirming and extending the SNP array findings. 1,3, deletion of 19q13 probe; 2, amplification of 13q34; 4, multiple amplifications of 13q34 probe; 5, amplification of 13q34 with monoallelic loss of the *RB1* gene; and 6, trisomy of chromosome 7

Mutation R132H in the *IDH1* gene was detected in all resections. The first resection was characterized by deletions, amplifications and CN-LOH on chromosomes 3, 7p, 9q, 12q, 13q and 19q (Fig. 2, Table 1). The changes in 13q and 19q were verified with I-FISH. No hypermethylation of the *MGMT* promoter or any other promoter analyzed was detected (average ratio ≥ 25 %) [8]. The second resection included the same CNVs as the first, with additional changes on 10p, 13q and 19p. The hypermethylation of the *MGMT* promoter was confirmed in this resection. Additional deletions at 8q11.1q22.3 and 10q22.3q23.1, and trisomy of chromosome 7 (7 % of cells), detected only with I-FISH, were found in the third resection.

**Table 1:** I-FISH, MLPA and SNP array findings for three lesions of case no. 1

	MLPA			MS-MLPA		I-FISH result	SNP array result	Treatment
	Gene	Location	Result	Gene	Result			
**First resection** - geminocystal astrocytoma	***IDH1***	**2q33.3**	**R132H**	*MSH2*	normal	deletion of 19q amplification of 13q	3p26.3p11.1(4,367,515-90,526,515)x1, 3q28q29(195,706,944-195,724,476)x1, 7p22.3p14.1(141,332-43,215,151)x2hmz, 12q12qq22(40,031,350-93,333,050)x1, 12q23.1q23.2(109,796,312-111,857,062)x1, 13q14.3(51,016,936-54,208,554)x1, 13q21.33q34(68,955,942-115,106,996)x3, 19q13.2q13.43(41,004,075-58,610,254)x1	NO
	***CRBN***	**3p26.3**	**del**					
	***CRELD1***	**3p25.3**	**del**					
	***VHL***	**3p25.3**	**del**	*MSH6*	normal			
	***PPARG***	**3p25.1**	**del**					
	***RAF1***	**3p25.1**	**del**					
	***HESX1***	**3p14.3**	**del**	*MLH1*	normal			
	*LFNG*	7p22.2	normal					
	*FKBP9*	7p14.3	normal					
	*GLI3*	7p14.1	normal	*MSH3*	normal			
	*ADAM22*	7q21.12	normal					
	*TFR2*	7q22.1	normal					
	*KIAA1549*	7q34	normal	*PMS2*	normal			
	*HIPK2*	7q34	normal					
	*MKRN1*	7q34	normal					
	*BRAF*	7q34	normal	*MGMT*	normal			
	*DPP6*	7q36.2	normal					
	*MIR31*	9p21.3	normal					
	*CDKN2A*	9p21.3	normal	*MLH3*	normal			
	*CDKN2B*	9p21.3	normal					
	*IDH2*	15q26.1	normal					
**Second resection** - diffuse astrocytoma	***IDH1***	**2q33.3**	**R132H**	*MSH2*	normal	deletion of 19q amplification of 13q	3p26.3p11.1(4,367,515-90,526,515)x1, 3q28q29(195,706,944-195,724,476)x1, 7p22.3p14.1(141,332-43,215,151) x2hmz, **9q34.11q34.3(131,306,847-138,275,572)x3,** **10p15.3(135,708-1,639,362)x3,** 12q12qq22(40,031,350-93,333,050)x1, 12q23.1q23.2(109,796,312-111,857,062)x1, 13q14.3(51,016,936-54,208,554)x1, **13q21.2q21.3 (61,757,866-62,558,596)x3,** **13q21.31(62,603,583-62,702,893)x1,** **13q21.31q21.33 (63,341,681-70,114,200)x1,** 13q21.33q34 (68,955,942-115,106,996)x3, 19q13.2q13.43(41,004,075-58,610,254)x1	NO
	***CRBN***	**3p26.3**	**del**					
	***CRELD1***	**3p25.3**	**del**					
	***VHL***	**3p25.3**	**del**	*MSH6*	normal			
	***PPARG***	**3p25.1**	**del**					
	***RAF1***	**3p25.1**	**del**					
	***HESX1***	**3p14.3**	**del**	*MLH1*	normal			
	*LFNG*	7p22.2	normal					
	*FKBP9*	7p14.3	normal					
	*GLI3*	7p14.1	normal	*MSH3*	normal			
	*ADAM22*	7q21.12	normal					
	*TFR2*	7q22.1	normal					
	*KIAA1549*	7q34	normal	*PMS2*	normal			
	*HIPK2*	7q34	normal					
	*MKRN1*	7q34	normal					
	*BRAF*	7q34	normal	***MGMT***	**methylated (51 %)**			
	*DPP6*	7q36.2	normal					
	*MIR31*	9p21.3	normal					
	*CDKN2A*	9p21.3	normal	*MLH3*	normal			
	*CDKN2B*	9p21.3	normal					
	*IDH2*	15q26.1	normal					
**Third resection** - anaplastic astrocytoma	***IDH1***	**2q33.3**	**R132H**	*MSH2*	normal	deletion of 19q amplification of 13q trisomy 7	3p26.3p11.1(4,367,515-90,526,515)x1, 3q28q29(195,706,944-195,724,476)x1, 7p22.3p14.1(141,332-43,215,151) x2hmz, **8q11.1q22.3 (48,043,253-106,052,343)x1,** 9q34.11q34.3(131,306,847-138,275,572)x3, 10p15.3 (135,708-1,639,362)x3, **10q22.3q23.1(81,643,451-84,775,286)x1,** 12q12qq22 (40,031,350-93,333,050)x1, 12q23.1q23.2(109,796,312-111,857,062)x1, 13q14.3 (51,016,936-54,208,554)x1, 13q21.2q21.3 (61,757,866-62,558,596)x3, 13q21.31 (62,603,583-62,702,893)x1, 13q21.31q21.33(63,341,681-70,114,200)x1, 13q21.33q34(68,955,942-115,106,996)x3, 19q13.2q13.43(41,004,075-58,610,254)x1	Chemotherapy
	***CRBN***	**3p26.3**	**del**					
	***CRELD1***	**3p25.3**	**del**					
	***VHL***	**3p25.3**	**del**	*MSH6*	normal			
	***PPARG***	**3p25.1**	**del**					
	***RAF1***	**3p25.1**	**del**					
	***HESX1***	**3p14.3**	**del**	*MLH1*	normal			
	*LFNG*	7p22.2	normal					
	*FKBP9*	7p14.3	normal					
	*GLI3*	7p14.1	normal	*MSH3*	normal			
	*ADAM22*	7q21.12	normal					
	*TFR2*	7q22.1	normal					
	*KIAA1549*	7q34	normal	*PMS2*	normal			
	*HIPK2*	7q34	normal					
	*MKRN1*	7q34	normal					
	*BRAF*	7q34	normal	***MGMT***	**methylated (51 %)**			
	*DPP6*	7q36.2	normal					
	*MIR31*	9p21.3	normal					
	*CDKN2A*	9p21.3	normal	*MLH3*	normal			
	*CDKN2B*	9p21.3	normal					
	*IDH2*	15q26.1	normal					

The pathogenetic findings for MLPA, MS-MLPA are marked with bold type. Abbreviation del means deleted. The SNP array results for each resection are notated according to the latest version of the International System of Human Cytogenetic Nomenclature (ISCN 2013) [17]. Findings described for first resection were found in all following samples therefore additional changes detected only in subsequent lesions are marked with bold type

**Fig. 3:**
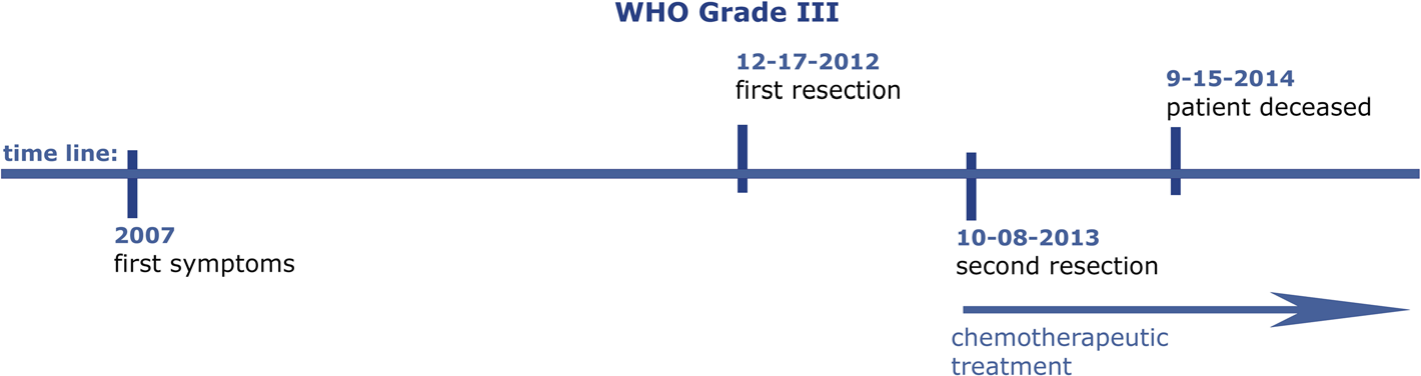
Time line representing the dates of resection and the type of treatment the case no. 2 received

Case no. 2 was a female patient diagnosed with recurrent anaplastic astrocytoma (WHO grade III) at the age of 36 years. The second resection (anaplastic astrocytoma, WHO grade III) was performed 10 months later (Fig. 3). The tumor was localized in left parietal lobe. The patient was treated with chemotherapy, but died 1 year after the second resection from further tumor progression. Patient´s survival was 87 months.

**Fig. 4:**
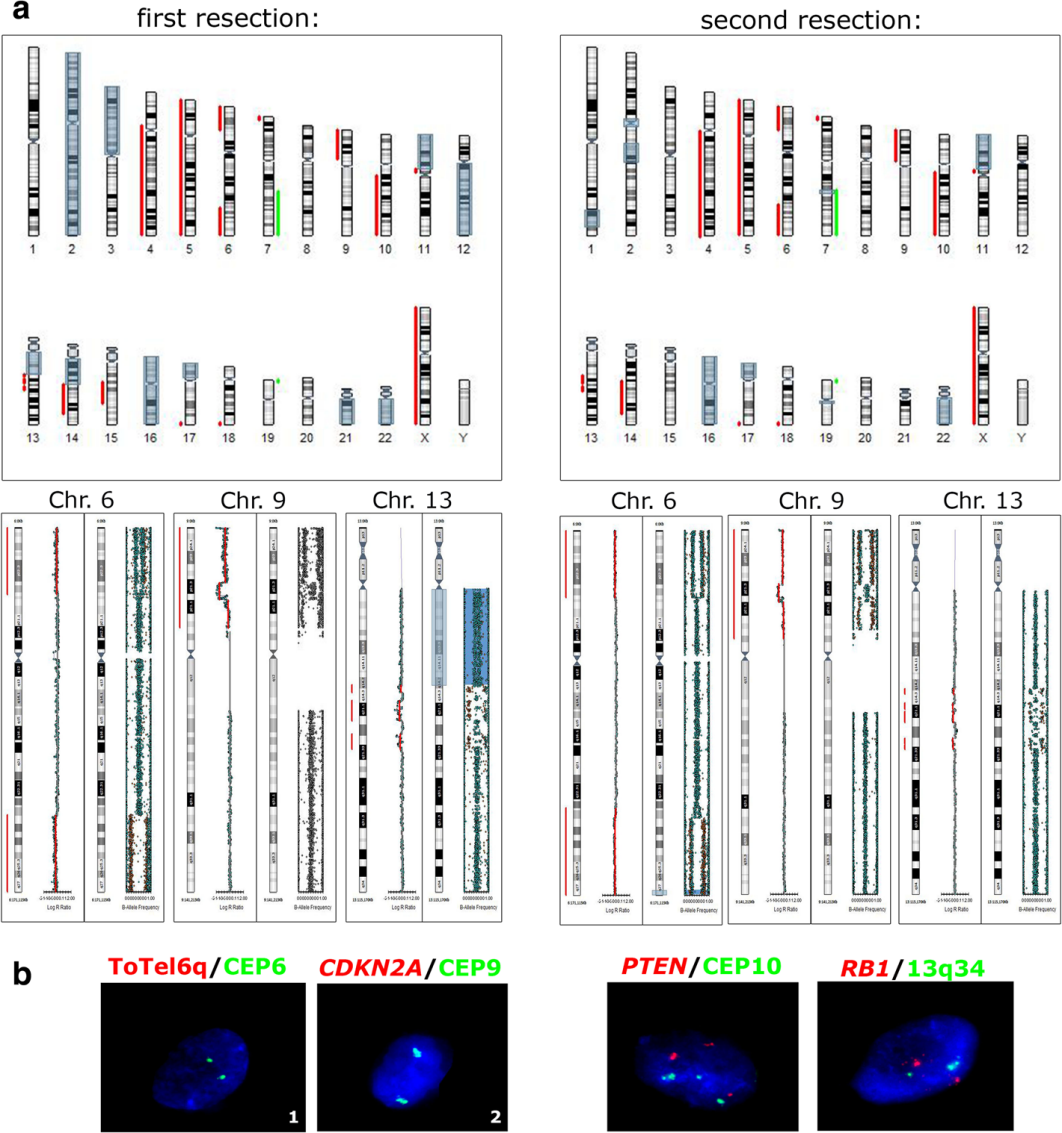
Molecular cytogenetic and epigenetic analyses of the three lesions of case no. 2. **a** Results of the SNP array for each lesion, with the selection of three chromosomes, in which the additional changes were detected. Red bars indicate the deletion of a chromosomal region. Green bars represent amplifications, and CN-LOH is marked with gray boxes. **b** Results of I-FISH analysis confirming and extending the SNP array findings. 1, biallelic deletion of subtelomeric area of the long arm of chromosome 6; 2, biallelic deletion of the *CDKN2A* gene; 3, deletion of the *PTEN* gene in polyploid nuclei; 4, the *RB1* gene and 13q34 were not affected by chromothripsis

**Fig. 5:**
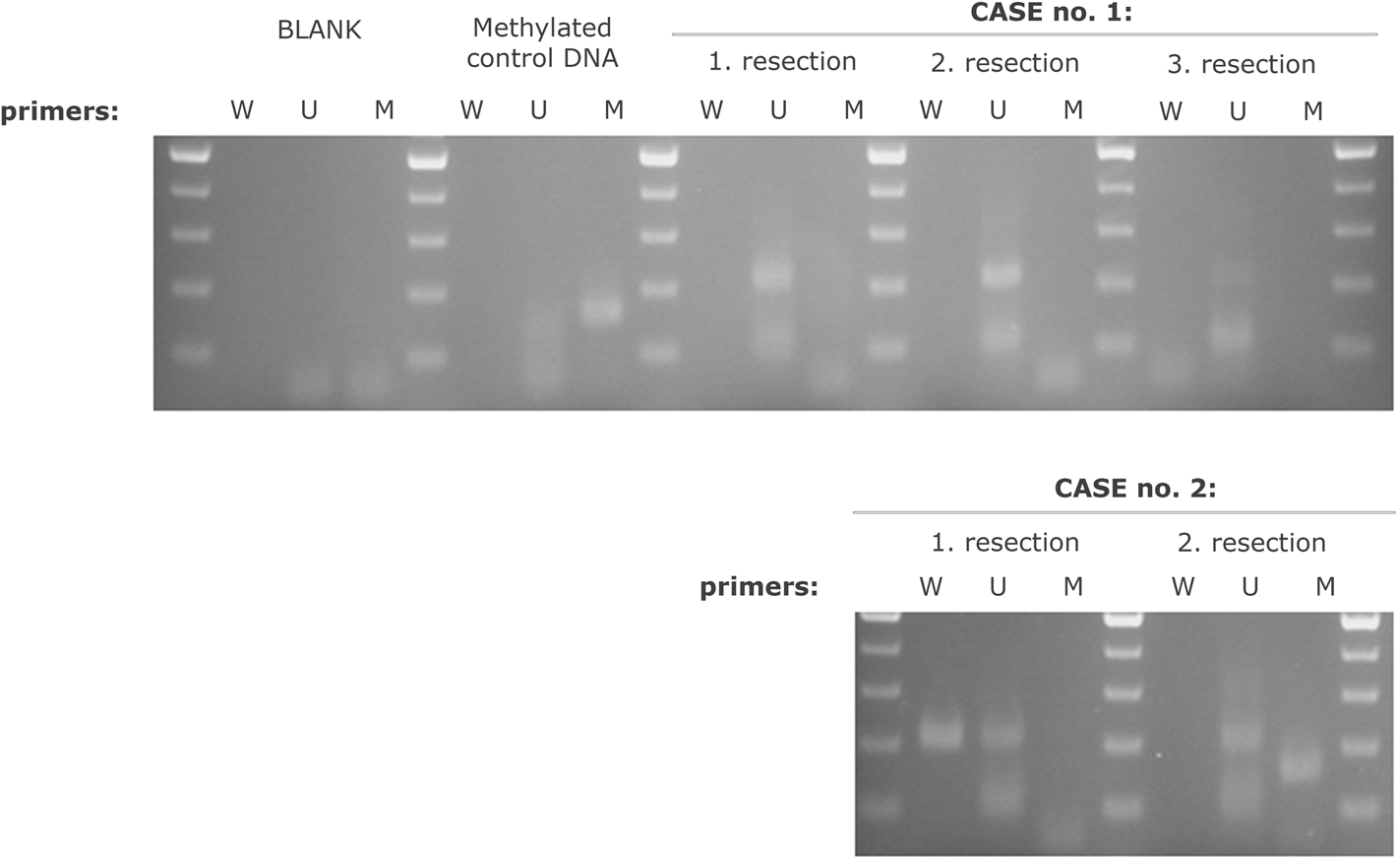
Verification of *MLH3* promoter methylation by MS-PCR. Three sets of primers designed to recognize unconverted DNA (W primers), converted unmethylated DNA (U primers), and converted methylated DNA (M primers) were used for each bisulfite-treated DNA

Both resections were characterized by the R132H mutation in the *IDH1* gene, CNVs including chromosomes X, 5, 6, 16, 17 and 22 and chromosomal arms 4q, 7q, 9p, 10q, 11p, 13q, 14q, 18q and 19p (Fig. 4, Table 2). A chromothripsis was also detected on chromosome arm 13q. The first specimen had several unique features: CN-LOH on chromosomes 2, 3p, 12q, and 21 and deletion of 15q. The second resection was defined by CN-LOH on 1q and 2q, and the hypermethylation of the *MLH3* and *MGMT* promoters. Methylation of *MHL3* promoter was verified by MS-PCR (Fig. 5). I-FISH analyses verified the deletion of *CDKN2A*, *PTEN* and 6q.

**Table 2:** I-FISH, MLPA and SNP array findings for two lesions of case no. 2

	MLPA			MS-MLPA		I-FISH	SNP array	Treatment
	Gene	Location	Result	Gene	Result			
**First resection** - anaplastic astrocytoma	***IDH1***	**2q33.3**	**R132H**	*MSH2*	normal	deletion of *CDKN2A* deletion of subtelomeric 16q	(X)x1, **(2)x2hmz,** **3p26.3p11.1(66,894-90,089,238)x2hmz,** 4q12q13.1(52,728,324-66,300,425)x1, (5)x1,6p25.3p21.33(204,909-31,335,647)x1, 6q22.33q27(128,989,302-170,898,549)x1, 7p22.3p22.1(46,239-5,016,400) x1, 7q22.1q36.3(98,451,358-159,119,486)x3, 9p24.3p13.1(46,587-38,771,460)x1, 10q21.2q26(61,465,713-135,430,043)x1, 11p15.5p11.2(203,788-47,430,599)x2hmz, 11p11.2 (47,801,101-51,274,692)x1, **12q12q24.33(38,416,139-133,429,634) x2hmz,** **13q12.11q14.2(19,888,741-50,144,613)x2hmz,** 13q14.2q21.33(50,283,115-69,338,113)cth, **14q11.2q22.2(20,213,937-54,316,607)x2hmz,** 14q23.2q32,12(62,420,606-93,152,220)x1, **15q21.1q24.1(46,805,269-74,506,930)x1,** (16)x2hmz, 17p13.3p11.2(18,901-20,219,226) x2hmz, 17q25.3(79,894,097-81,047,565)x1, 18q23(76,324,504-78,014,582)x1, 19p13.3 (267,039-1,569,839)x3, **(21)x2hmz,** (22)x2hmz	Chemotherapy
	*CRBN*	3p26.3	normal					
	*CRELD1*	3p25.3	normal					
	*VHL*	3p25.3	normal	*MSH6*	normal			
	*PPARG*	3p25.1	normal					
	*RAF1*	3p25.1	normal					
	*HESX1*	3p14.3	normal	*MLH1*	normal			
	***LFNG***	**7p22.2**	**del**					
	*FKBP9*	7p14.3	normal					
	*GLI3*	7p14.1	normal	*MSH3*	normal			
	*ADAM22*	7q21.12	normal					
	*TFR2*	7q22.1	normal					
	***KIAA1549***	**7q34**	**amp**	*PMS2*	normal			
	***HIPK2***	**7q34**	**amp**					
	***MKRN1***	**7q34**	**amp**					
	***BRAF***	**7q34**	**amp**	*MGMT*	methylated (19 %)			
	***DPP6***	**7q36.2**	**amp**					
	***MIR31***	**9p21.3**	**del**					
	***CDKN2A***	**9p21.3**	**del**	*MLH3*	normal			
	***CDKN2B***	**9p21.3**	**del**					
	*IDH2*	15q26.1	normal					
**Second resection** - anaplastic astrocytoma	***IDH1***	**2q33.3**	**R132H**	*MSH2*	normal	deletion of *CDKN2A* deletion of PTEN	(X)x1, **1q41q43(216,318,021-241,388,129) x2hmz,** **2q14.2q22.3(120,617,488-144,598,432) x2hmz,** 4q12q13.1 (52,728,324-66,300,425)x1, (5)x1,6p25.3p21.33 (204,909-31,335,647)x1, 6q22.33q27(128,989,302-170,898,549)x1, 7p22.3p22.1 (46,239-5,016,400)x1, 7q22.1q36.3(98,451,358-159,119,486)x3, 9p24.3p13.1 (46,587-38,771,460)x1, 10q21.2q26(61,465,713-135,430,043)x1, 11p15.5p11.2 (203,788-47,430,599)x2hmz, 11p11.2(47,801,101-51,274,692)x1, 13q14.2q21.33 (50,283,115-69,338,113)cth, 14q23.2q32,12 (62,420,606-93,152,220)x1, (16)x2hmz, 17p13.3p11.2(18,901-20,219,226)x2hmz, 17q25.3(79,894,097-81,047,565)x1, 18q23(76,324,504-78,014,582)x1, 19p13.3(267,039-1,569,839)x3, (22)x2hmz	Chemotherapy
	*CRBN*	3p26.3	normal					
	*CRELD1*	3p25.3	normal					
	*VHL*	3p25.3	normal	*MSH6*	normal			
	*PPARG*	3p25.1	normal					
	*RAF1*	3p25.1	normal					
	*HESX1*	3p14.3	normal	*MLH1*	normal			
	***LFNG***	**7p22.2**	**del**					
	*FKBP9*	7p14.3	normal					
	*GLI3*	7p14.1	normal	*MSH3*	normal			
	*ADAM22*	7q21.12	normal					
	*TFR2*	7q22.1	normal					
	***KIAA1549***	**7q34**	**amp**	*PMS2*	normal			
	***HIPK2***	**7q34**	**amp**					
	***MKRN1***	**7q34**	**amp**					
	***BRAF***	**7q34**	**amp**	***MGMT***	**methylated (25 %)**			
	***DPP6***	**7q36.2**	**amp**					
	***MIR31***	**9p21.3**	**del**					
	***CDKN2A***	**9p21.3**	**del**	***MLH3***	**methylated**			
	***CDKN2B***	**9p21.3**	**del**					
	*IDH2*	15q26.1	normal					

The pathogenetic findings for MLPA, MS-MLPA and I-FISH are marked with bold type. Abbreviation del means deleted, amp is amplificated. The SNP array results for each resection are notated according to the latest version of the International System of Human Cytogenetic Nomenclature (ISCN 2013) [17]. Aberrations unique to each resection are marked with bold text

## Discussion

Gliomas represent typical examples of multistep oncogenesis, in which new mutations are acquired under clonal selection, and the tumor can thus evolve to a more aggressive form [9]. Both our cases were characterized by a set of CNVs that was present in all the samples acquired for each patient, even after the radical resection of the previous lesion (Figs. 2, 4 and Tables 1, 2). However, whereas case no. 1 showed the typical acquisition of genetic and epigenetic aberrations in each step of the evolution of his astrocytoma, case no. 2 was characterized by different additional changes in each resection. These findings support the theory of the monoclonal origin of astrocytomas, and also suggest that a specific set of genetic features, typical for each patient, are necessary to maintain the glial cells in the tumor state [9, 10].

The *IDH1* gene was mutated in both primary lesions. The mutated form of the *IDH1* gene leads to the epigenetic deregulation described as the ‘glioma CpG island methylator phenotype’ [11]. Therefore, the *MGMT* hypermethylation observed in both cases and the methylation of the *MLH3* promoter in case no. 2 might result from the mutated *IDH1* gene. The methylation of both promoters always occurred as a secondary event in the recurrent lesion, so our finding supports the hypothesis that *IDH1* mutation is the primary event in glioma carcinogenesis [12]. The methylation of the *MLH3* promoter is a new finding, and is reported to appear in 27 % of astrocytomas [7], although its role in glioma is yet to be investigated.

Chromothripsis was recently described in gliomas with *IDH1* gene mutation. Although no prognostic significance was observed in that study [13], it is generally believed that chromothripsis contributes to tumorigenesis. We observed chromothripsis on 13q in the astrocytoma of patient no. 2, who died after the third resection from tumor progression. Whether chromothripsis was one of the triggers of tumor progression remains to be clarified.

Progression towards a higher WHO grade occurred between the second and third resection in case no. 1. The additional aberrations that occurred between these lesions were deletions at 8q11.1q22.3, which contains 166 OMIM genes, and at 10q22.3q23.1 where 13 genes are localized. Two of these genes are neuregulin 3 (*NRG3*, 10q23.1), which encodes an oligodendrocyte survival factor [14], and nibrin (*NBN*, 8q21.3), which encodes a protein crucial for maintaining genomic stability by affecting the DNA damage signaling pathway [15]. Therefore, the deletion of these genes might influence the tumorigenic potential of astrocytoma.

## Conclusions

Our data suggest that each of investigated astrocytomas share a set of CNVs and epigenetic modifications that are necessary for maintaining the malignant status of glial cells during tumor evolution. However, new genetic or epigenetic markers, such as deletions on 8q,10q or the methylation of the *MLH3* promoter, may occur in response to clonal selection. The emergence of new aberrations, caused by treatment or of random gains that improve clonal proliferation may also influence the patient’s response to treatment. MutS homolog 6 (*MSH6*) may be one such example. Its mutation is induced by temozolomide treatment and causes drug resistance in the affected glioblastomas [16].

Therefore, each tumor recurrence must be genetically and epigenetically characterized to allow the correct therapeutic decision to be made. The methylation of the *MLH3* promoter, the deletion of the *NGR3* and *NBN* genes or chromothripsis on 13q observed in our study are potential phenomena that might influence tumor cell behavior and thus modulate the tumor’s responsiveness to treatment.
